# Relationship between TRAIL and Left Ventricular Ejection Fraction in Patients with ST-Elevation Myocardial Infarction Treated with Primary Percutaneous Coronary Intervention

**DOI:** 10.1155/2018/3709084

**Published:** 2018-07-09

**Authors:** Elena Teringova, Martin Kozel, Jiri Knot, Viktor Kocka, Klara Benesova, Petr Tousek

**Affiliations:** ^1^Cardiocenter, Department of Cardiology, 3rd Faculty of Medicine, Charles University and University Hospital Kralovske Vinohrady, Prague, Czech Republic; ^2^Institute for Biostatistics and Analyses of The Faculty of Medicine, Faculty of Medicine, Masaryk University, Brno, Czech Republic

## Abstract

**Background:**

Apoptosis plays an important role in the myocardial injury after acute myocardial infarction and in the subsequent development of heart failure.

**Aim:**

To clarify serum kinetics of apoptotic markers TRAIL and sFas and their relation to left ventricular ejection fraction (LVEF) in patients with ST-elevation myocardial infarction (STEMI) treated with primary percutaneous coronary intervention (pPCI).

**Methods:**

In 101 patients with STEMI treated with pPCI, levels of TRAIL and sFas were measured in series of serum samples obtained during hospitalization and one month after STEMI. LVEF was assessed at admission and at one month. Major adverse cardiovascular events (MACE, i.e., death, re-MI, and hospitalization for heart failure and stroke) were analysed during a two-year followup.

**Results:**

Serum level of TRAIL significantly decreased one day after pPCI (50.5pg/mL) compared to admission (56.7pg/mL), subsequently increased on day 2 after pPCI (58.8pg/mL), and reached its highest level at one month (70.3pg/mL). TRAIL levels on days 1 and 2 showed a significant inverse correlation with troponin and a significant positive correlation with LVEF at baseline. Moreover, TRAIL correlated significantly with LVEF one month after STEMI (day 1: r=0.402, p<0.001; day 2: r=0.542, p<0.001). On the contrary, sFas level was significantly lowest at admission (5073pg/mL), increased one day after pPCI (6370pg/mL), and decreased on day 2 (5548pg/mL). Significantly highest sFas level was marked at one month (7024pg/mL). sFas failed to correlate with LVEF at baseline or at one month. Both TRAIL and sFas showed no ability to predict improvement of LVEF one month after STEMI or a 2-year MACE (represented by 3.29%).

**Conclusion:**

In STEMI treated with pPCI, TRAIL reaches its lowest serum concentration after reperfusion. Low TRAIL level is associated with worse LVEF in the acute phase of STEMI as well as one month after STEMI. Higher TRAIL level appears to be beneficial and thus TRAIL seems to represent a protective mediator of post-AMI injury.

## 1. Introduction

Acute myocardial infarction (AMI) represents a major cause of morbidity and mortality worldwide. Despite significant improvement in the treatment of AMI in the past decades, many patients subsequently suffer from left ventricular (LV) dysfunction and heart failure. Post-AMI heart failure represents a high-risk condition with a poor long-term prognosis [1, 2]. Apoptosis plays an important role in the myocardial loss after AMI, as well as in the process of LV remodelling and development of heart failure [3–5]. Thus recognizing a sensitive apoptotic marker that would help in prognostic stratification of AMI patients is of a great importance.

TNF-related apoptosis-stimulating ligand (TRAIL) and apoptosis-stimulating fragment (sFas) are both soluble apoptotic markers that can induce apoptosis [6, 7]. After binding to their receptors (TRAIL to its receptors TRAIL-R1 and TRAIL-R2, sFas to its Fas receptor), apoptosis is induced through death-receptor signaling pathway, resulting in caspase-8 activation, which activates executioner caspase-3 and triggers the terminal phase of apoptosis [8, 9].

Levels of soluble sFas and TRAIL were assessed in population of AMI patients and heart failure patients to test their ability to predict prognosis [10–15]. Higher sFas levels in heart failure patients were associated with higher risk of mortality and rehospitalization for heart failure [12–14]. Concerning TRAIL, lower TRAIL levels were associated with poor prognosis in heart failure patients and in elderly patients with cardiovascular disease [14, 15]. In acute coronary syndrome patients, decreased TRAIL levels were found to represent a significant predictor of mortality and hospitalization for heart failure [11].

The aim of the present study was to assess levels of both TRAIL and sFas in a homogenous group of patients with ST-elevation myocardial infarction (STEMI) treated with primary percutaneous coronary intervention (pPCI) in series of serum samples obtained during hospitalization and at one-month followup, to clarify the kinetics of serum levels of the two abovementioned apoptotic markers after STEMI. Since apoptosis represents an important contributor to cardiomyocyte loss after AMI (initiated during the ischemic insult, subsequent reperfusion injury as well as within the process of ventricular remodelling) [3–5, 16, 17], we aimed to test the correlation between levels of apoptotic markers and LV ejection fraction (LVEF) after STEMI. Furthermore, we aimed to determine whether levels of TRAIL and sFas relate to LVEF change during one-month followup. Lastly, we aimed to validate their prognostic significance during 2-year clinical followup.

## 2. Methods

### 2.1. Study Population and Followup

Study participants were prospectively enrolled in the Cardiocenter at the University Hospital Kralovske Vinohrady, Prague, from December 2012 to June 2014. The inclusion criterion was STEMI treated using primary percutaneous coronary intervention (pPCI). Diagnosis was made based on typical ischemic symptoms and changes in electrocardiogram (ECG) according to the guidelines of the European Society of Cardiology for the management of STEMI [18]. The exclusion criteria were as follows: (1) no revascularisation possible, (2) life expectancy less than one year due to noncardiac reasons, and (3) reluctance to cooperate in a long-term project. Echocardiographic examination was performed in all patients on the first day of hospitalization for STEMI. The study complies with the Declaration of Helsinki and was approved by the local Ethics Committee. Each patient signed written informed consent.

Followup visits including echocardiographic examination were arranged one month after the index procedure at the outpatient department. Patients were further followed for two years for mortality and morbidity endpoints either by clinical controls or telephonically.

### 2.2. Blood Sampling and Laboratory Analysis

Apoptotic markers were analysed from venous blood samples obtained from each patient at four different time points: at admission-prior to pPCI (day 0), 24 hours +/- 6 hours after pPCI (day 1), two days after pPCI (day 2), and at a 30-day control. After centrifugation (3500 rpm, 15 min), serum was stored at -70°C. Commercially available Enzyme-Linked Immuno-Sorbent Assays (ELISA) were used to measure serum concentrations of the reported apoptotic markers (sFas and TRAIL - R&D Systems, Minneapolis, MN, USA). Intra- and interassay coefficients were 4.60% and 6.70% for sFas and 5.60% and 7.40% for TRAIL. The lowest concentration detectable was 20pg/mL for sFas and 7.87pg/ml for TRAIL. All measurements were performed by staff unaware of the clinical data.

High-sensitive cardiac troponin T (hs-cTnT) was measured by Roche assay at admission and one and two days after pPCI—at the same time points as assessment of levels of apoptotic markers. Blood samples for biochemistry and haematology tests were taken at admission.

### 2.3. Clinical and Echocardiographic Evaluation

Echocardiographic examination was performed in all patients on the first day of hospitalization and at one-month clinical followup. A standard echocardiographic imaging protocol was used with the apical 4- and 2-chamber views and long and short parasternal axis views. The left ventricular ejection fraction (LVEF) was evaluated by using the biplane modified Simpson rule. To limit the variation, final LVEF was determined as a result of two examiners consensus. All echocardiographic examinations were analysed at the Echocardiographic Laboratory of the Cardiocenter at the University Hospital in Kralovske Vinohrady, Prague.

Patients were followed for two years after the index event and major adverse cardiovascular events (i.e., death, re-IM, hospitalization for heart failure, and stroke) were analysed.

### 2.4. Statistical Analysis

Continuous data were tested for distribution using the Kolmogorov-Smirnov test. Continuous data with normal distribution are presented as mean ± SD, with non-Gaussian distribution as median (interquartile range). Statistical comparison of change in apoptotic markers within individual patients was done using Friedman test and Kendall's W, post hoc analysis was performed using Wilcoxon signed-rank tests with Bonferroni correction. Relation between continuous values was described using Pearson's correlation coefficient and its significance (both crude and adjusted for confounding factors). Potential confounding factors which were taken into consideration: age, gender, BMI, presence of diabetes mellitus, arterial hypertension, Killip class, and infarct-related artery. Predictive power of analysed markers for the improvement of LVEF was analysed using ROC analysis and described by its AUC and specificity and sensitivity at cut-off. The ability of TRAIL and sFas to predict a 2-year MACE was analysed using logistic regression. Two-tailed p value of less than 0.05 was considered to be significant; statistical analysis was computed using SPSS 22.0.0.1 (IBM Corporation, 2014).

## 3. Results

### 3.1. Baseline Characteristics

A total of one hundred and fifteen patients were enrolled in the study. A one-month followup was achieved in one hundred and one patients (87.8%). Baseline characteristics of the study population are summarized in Table 1.

### 3.2. Dynamic Changes in Serum Levels of TRAIL and sFas after STEMI

**Figure 1:**
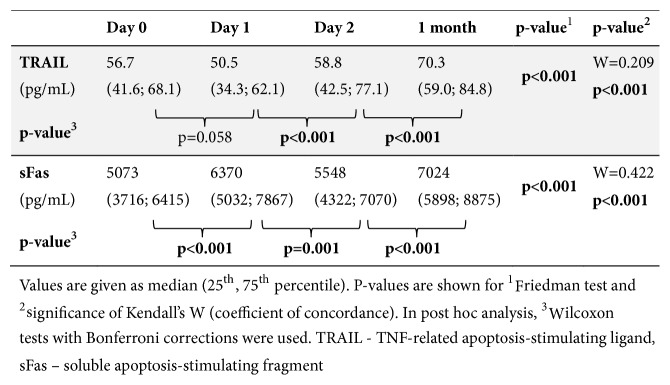
Serum concentrations of soluble TRAIL and sFas.

Serum levels of TRAIL and sFas measured in STEMI patients during hospitalization and at a 1-month followup are summarized in Figure 1.

Concerning TRAIL, its level decreased one day after pPCI compared to admission level (day 0). TRAIL subsequently increased on day 2 and reached its highest level measured in our study at 1 month (Figure 1).

On the contrary, sFas level increased one day after pPCI compared to admission. sFas subsequently decreased on day 2 and second rise of sFas was marked at 1 month (Figure 1).

All changes of sFas and TRAIL levels within individual patients were statistically significant.

### 3.3. Correlation between Markers of Apoptosis and Necrosis

**Figure 2:**
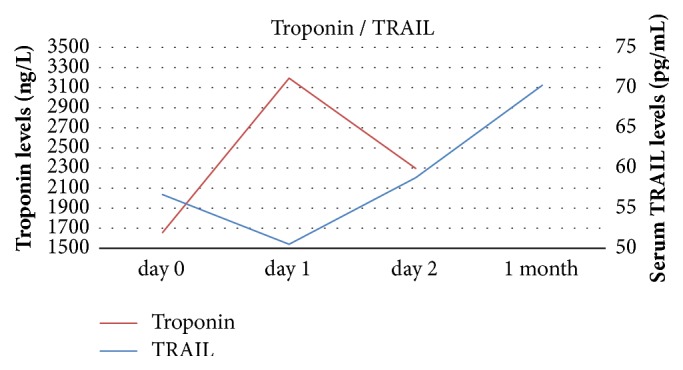
Relationship between serum level of TRAIL and hs-cTnT.

**Figure 3:**
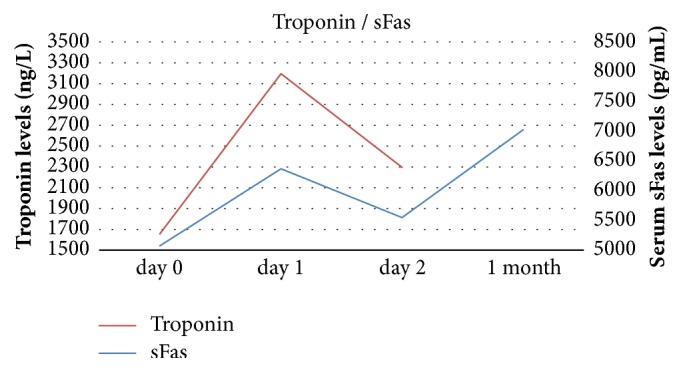
Relationship between serum level of sFas and hs-cTnT.

Statistical analysis showed a significant negative correlation between levels of TRAIL and troponin on days 1 and 2 after pPCI. sFas levels correlated with troponin only on day 2 after pPCI and the correlation was bordering on the statistical significance. Results are summarized in Table 2. Relationships between troponin and apoptotic markers TRAIL and sFas are visualised in Figures 2 and 3.

### 3.4. Correlation between Markers of Apoptosis and Time to pPCI

There was a negative correlation between time from the onset of symptoms to pPCI and level of TRAIL at admission (day 0: r= - 0.33, p=0.002; day 1: r= -0.19, p=0.08). No correlation was found between time to pPCI and sFas levels.

### 3.5. Correlation between Markers of Apoptosis and LVEF

Among 101 patients who completed a 1-month followup, echocardiographic examination was available in 94 patients. Mean LVEF at baseline was 47.25% ± 8.82. One month after STEMI, mean LVEF improved to 55.78% ± 8.96, which represents an average improvement of 8.62% ± 8.16. One month after STEMI, improvement of LVEF ≥ 10% was present in 51 patients.

Statistical analysis showed a positive correlation between levels of TRAIL and LVEF at baseline—results are summarized in Table 3. Moreover, TRAIL levels on days 1 and 2 correlated positively also with LVEF at 1 month (Table 3). There was no correlation found between sFas levels and LVEF at baseline or at 1 month.

Apoptotic markers were further tested for their ability to predict improvement of LVEF. However, receiver-operating characteristic curve analysis showed that neither TRAIL nor sFas were able to predict improvement of LVEF ≥10% one month after STEMI. Similarly to apoptotic markers, also troponin failed to predict improvement of LVEF one month after STEMI. Results are shown in Tables 4 and 5.

### 3.6. Two-Year Followup

A two-year followup was achieved in 91 patients (90%). Major adverse cardiovascular events were present in 3 patients, which represents 3.3%. One patient had died, two patients had had re-MI, no one had been hospitalized for heart failure, and no one had experienced stroke.

## 4. Discussion

In our study, we demonstrated how serum levels of soluble TRAIL and sFas evolve after STEMI treated with pPCI. TRAIL decreased one day after pPCI compared to admission and then progressively increased on day 2 and reached its highest level measured in our study at one month. Our findings confirm and extend recently published studies, which have demonstrated that TRAIL level is significantly decreased in AMI patients [10, 11]. Our results provide a detailed description of how TRAIL serum level ranges in the acute phase of STEMI as well as one month after STEMI.

TRAIL represents a promising marker of prognosis in AIM patients and is considered a protective mediator in post-AMI injury. Lower TRAIL level is associated with worse patient prognosis while higher TRAIL level seems to be protective [10, 11]. Secchiero et al. measured TRAIL in a population of 60 AMI patients and demonstrated that TRAIL levels were significantly lower at admission for AMI compared to healthy controls, increased at discharge, and normalized at 6-12 months [10]. In our study, more detailed examination of the first three days of STEMI showed that TRAIL reached its minimum one day after pPCI and then progressively increased. Decrease in TRAIL level 24 hours after pPCI could be related to reperfusion injury. Reperfusion injury with enhanced inflammatory reaction is associated with increased level of many cytokines and proteolytic enzymes, such as matrix metalloproteinases [19]. Metalloproteinase 2 was shown to have the ability to cleave recombinant TRAIL in vitro [20]. Thus degradation of TRAIL by proteolytic enzymes released at reperfusion, such as metalloproteinase 2, could represent one of potential explanations for decreased TRAIL level after PCI. The exact molecular mechanism of TRAIL's function, however, has not yet been completely understood. In tumor cell lines, TRAIL binds to its receptors (TRAIL receptors 1 and 2) and initiates intracellular signaling cascade resulting in the apoptotic cell death [8, 9]. The effect of TRAIL on normal cells is yet unclear. Some authors reported that TRAIL-induced apoptosis could be specific to cancer cells, sparing the normal cells [21], while others described that TRAIL can induce apoptosis also in normal human hepatocytes and endothelial cells [22, 23]. TRAIL has also been referred to as a modulator of inflammatory response [24] and some experimental data suggest that TRAIL receptors 1 and 2 can also mediate cell type-dependent prosurvival and proliferation signals [25]. In a diabetic mouse model, direct administration of TRAIL reduced development of cardiomyopathy [26] and another similar study in diabetic mice demonstrated that systemic TRAIL delivery exhibited antiatherosclerotic activity [27]. Despite undetermined function of TRAIL at the molecular level, in clinical studies, lower TRAIL levels have been associated with worse patient prognosis while higher levels of TRAIL seem to be protective. Thus inhibition of TRAIL degradation or/and an enhancement of TRAIL availability could represent an interesting field of investigation and a potential target of therapeutic intervention.

Our study also showed that TRAIL level at admission correlated inversely with the time from the onset of symptoms to pPCI. The trend also continued on the 1st day after pPCI. The longer the ischemic insult, lower the level of TRAIL and the higher the level of troponin. Osmancik et al. examined TRAIL level in 295 acute coronary syndrome patients and followed them for 6 months. Low TRAIL level was the strongest significant and independent predictor of death and hospitalization for heart failure [11]. In line with Osmancik results, TRAIL in our study correlated significantly with important prognostic markers: inversely with concentration of troponin and positively with LVEF. Moreover, our study showed a significant positive correlation between TRAIL and LVEF one month after STEMI. These findings support TRAIL as a protective mediator in post-AMI injury. However, TRAIL failed to have the ability to predict improvement of LVEF at one month. We assume this failure could be explained by the size and the spectrum of our study group. Small sample size and a selected group of patients according to their willingness to cooperate in a long-term project could have influenced the results. TRAIL levels in our study group were generally higher compared to Osmancik's study [11]. There, TRAIL concentration of 44.6pg/mL at admission was identified as a cut-off value for prediction of poor prognosis. TRAIL level at admission in our study group was 56.7pg/mL. These results also correspond with a small number of endpoints during our two-year followup. Similarly to TRAIL, also troponin failed to predict improvement of LVEF one month after STEMI in our study, even though some recent studies reported troponin as an important predictor of LVEF after STEMI [28, 29]. Thus larger study group could have provided a better understanding of TRAIL's role in LVEF recovery after STEMI. Additionally, evolution of post-AMI LVEF represents a multifactorial process influenced by several other cofactors besides cardiomyocyte loss (such as the extend of stunned myocardium, function of myocardial microvascular circulation, level of oxidative stress, inflammatory response, extracellular matrix alterations, etc.). As a result, simple assessment of markers of apoptosis might not reach the ability to predict improvement of LVEF after AMI.

Concerning sFas levels, previous studies demonstrated elevated sFas levels in patients with acute myocardial infarction [30, 31]. However, sFas levels failed to correlate with infarct size [30], measures of LV remodelling [31], or patient prognosis [11]. These findings were confirmed also in a study with pure STEMI population—Nilsson et al. measured sFas levels in 48 STEMI patients prior to PCI and 24 hours after the procedure and used cardiac MRI to assess infarct size and parameters of LV dysfunction and remodelling at 5 days and 4 months after STEMI [32]. sFas levels did not show any consistent correlation with any of the measured parameters. Interestingly, level of sFas at 24 hours after PCI was significantly higher than sFas measured at admission. In concordance with Nilsson's results, sFas levels measured in our study behaved similarly. sFas level increased significantly one day after pPCI compared to baseline. Serum samples obtained at later time points revealed that sFas level significantly decreased two days after pPCI and increased again at one month. However, sFas showed no correlation with LVEF at baseline or 1 month after STEMI. Increase in the serum level of sFas after AIM is a result of release from myocardial tissue [31], however, the role of Fas-mediated apoptosis in post-AMI injury remains yet undetermined. Studies with heart failure patients demonstrated association of increased sFas levels with worse patient prognosis [12–14]. In our STEMI group, the highest sFas levels were measured 1 month after STEMI, but still sFas levels were dramatically lower compared to sFas levels reported in high-risk heart failure patients [14]. Measuring sFas levels in the acute phase of AIM that are significantly lower and without prognostic value thus seems to be inefficient.

In conclusion, our results demonstrate how serum levels of TRAIL and sFas evolve in STEMI patients treated with pPCI. TRAIL decreases one day after pPCI compared to admission, then increases on day 2, and reaches its highest level measured in our study one month after STEMI. TRAIL levels show significant inverse correlation with troponin levels and with time-to-pPCI interval. TRAIL correlates positively with LVEF at baseline as well as with LVEF one month after STEMI. Low TRAIL levels are associated with worse LVEF after STEMI. Thus TRAIL seems to be a protective mediator of post-AMI injury. On the contrary, sFas level increased one day after pPCI compared to admission, then decreased on day 2, and increased again one month after STEMI. sFas failed to correlate with LVEF at baseline or at one month. The role of sFas in post-AMI injury is yet uncertain.

## Figures and Tables

**Table 1:** **Baseline characteristics** of the study population including medical history, index event and angiography characteristics and medication at discharge (n = 101).

**Baseline characteristics**	
Age, years (mean, SD)	59.36 ± 10.00
Male gender (n, %)	75 (74.3)
BMI (mean, SD)	28.00 ± 4.07
DM (n, %)	17 (16.8)
Hypertension (n, %)	53 (52.5)
Smoking status (n, %)	82 (81.2)
History of MI (n, %)	10 (9.9)

**Index event and angiography characteristics**	
Time-to-PCI, minutes (median, 25th, 75th percentile)	180 (120, 370)
**Killip class**	
Killip class I-II	100 (99.0)
Killip class III	1 (1.0)
CAD severity (mean, SD)	1.85 ± 0.79
**Infarct related artery**	
LAD (n, %)	41 (40.6)
LCx (n, %)	16 (15.8)
RCA (n, %)	44 (43.6)
**Type of stent**	
BMS (n, %)	16 (15.8)
DES (n, %)	32 (31.7)
Absorb (n, %)	49 (48.5)
TIMI flow 3 after PCI (n, %)	98 (97)
Complete revascularization (n, %)	60 (60.0)

**Medication at discharge**	
Beta-blocker (n, %)	92 (91.1)
ACE inhibitor (n, %)	90 (89.1)
Aspirin (n, %)	96 (95.1)
Statin (n, %)	99 (98.0)
Clopidogrel (n, %)	18 (17.8)
Prasugrel (n, %)	48 (47.5)
Ticagrelor (n, %)	35 (34.7)

BMI – body mass index, DM – the presence of diabetes mellitus, smoking status – smoking before admission, MI – myocardial infarction, time-to-pPCI – time from the onset of symptoms to primary percutaneous coronary intervention, LAD – left anterior descending artery, LCx – left circumflex artery, RCA – right coronary artery, BMS – bare metal stent, DES – drug eluting stent, Absorb – bioresorbable stent, TIMI flow – “thrombolysis in myocardial infarction” grade flow, complete revascularization – the absence of any stenosis of 60% or more in at least one coronary artery at discharge.

**Table 2:** The correlation between markers of apoptosis and troponin.

	**r**	**p-value**	**r (adj.)**	**p-value**
**Correlation between TRAIL and hs-cTnT **				
Day 0	-0.106	0.299	-0.062	0.561
Day 1	**-0.387**	**<0.001**	**-0.379**	**<0.001**
Day 2	**-0.486**	**<0.001**	**-0.510**	**<0.001**

**Correlation between sFas and hs-cTnT**				
Day 0	0.127	0.216	0.152	0.154
Day 1	-0.049	0.639	-0.019	0.861
Day 2	-0.225	0.054	**-0.249**	**0.042**

Correlation is described using Pearson's correlation coefficient and its significance (both crude and adjusted for confounding factors). TRAIL: TNF-related apoptosis-stimulating ligand and hs-cTnT: high sensitive cardiac troponin T.

**Table 3:** The correlation between TRAIL and LVEF at baseline and at 1 month (described using Pearson's correlation coefficient and its significanc —crude and adjusted for confounding factors).

	**r**	**p-value**	**r (adj.)**	**p-value**
**Correlation between TRAIL and LVEF at baseline**				
Day 0	**0.252**	**0.013**	0.168	0.113
Day 1	**0.301**	**0.003**	**0.320**	**0.002**
Day 2	**0.455**	**<0.001**	**0.554**	**<0.001**

**Correlation between TRAIL and LVEF at 1-month**				
Day 0	0.158	0.136	0.076	0.495
Day 1	**0.368**	**<0.001**	**0.302**	**0.006**
Day 2	**0.505**	**<0.001**	**0.398**	**0.001**

**Table 4:** The correlation between troponin and LVEF at baseline and at 1 month (described using Pearson's correlation coefficient and its significance—crude and adjusted for confounding factors).

	**r**	**p-value**	**r (adj.)**	**p-value**
**Correlation between hs-cTnT and LVEF at baseline**				
Day 0	**-0.284**	**0.005**	**-0.287**	**0.007**
Day 1	**-0.550**	**<0.001**	**-0.542**	**<0.001**
Day 2	**-0.613**	**<0.001**	**-0.612**	**<0.001**

**Correlation between hs-cTnT and LVEF at 1-month**				
Day 0	**-0.460**	**<0.001**	**-0.448**	**<0.001**
Day 1	**-0.513**	**<0.001**	**-0.520**	**<0.001**
Day 2	**-0.656**	**<0.001**	**-0.690**	**<0.001**

**Table 5:** Ability of apoptotic markers to predict improvement of LVEF ≥10%. (predictive power of analysed markers for the improvement of LVEF was analysed using ROC analysis and described by its AUC).

	**AUC (95% CI)**	**p-value**
**TRAIL**		
Day 0	55.0 (42.7; 67.2)	0.421
Day 1	50.2 (37.7; 62.8)	0.970
Day 2	57.9 (45.0; 70.8)	0.220

**sFas**		
Day 0	53.2 (41.1; 65.3)	0.606
Day 1	54.6 (42.5; 66.7)	0.455
Day 2	55.9 (43.4; 68.4)	0.362

**hs-cTnT**		
Day 0	54.0 (34.1; 57.8)	0.502
Day 1	53.8 (41.6; 66.0)	0.533
Day 2	50.0 (37.0; 63.0)	0.999
Peak hs-cTnT	52.9 (40.7; 65.0)	0.633

## Data Availability

The data used to support the findings of this study are available from the corresponding author upon request.

## Additional Points

*Study Limitations*. Our study limitations are related mainly to sample size, patient selection, followup completion, and absence of a control group. Patients participating in the study were not enrolled consecutively but selected, due to better coordination with the project from a long-term prospective. Also, one month followup was not achieved in 12.2% of patients, of who a 2-year followup was not completed in 10%. These limitations could have influenced our results.

## References

[1] Chen J., Hsieh A. F.-C., Dharmarajan K., Masoudi F. A., Krumholz H. M. (2013). National trends in heart failure hospitalization after acute myocardial infarction for medicare beneficiaries 1998-2010. *Circulation*.

[2] Hung J., Teng T. K., Finn J. (2013). Trends From 1996 to 2007 in Incidence and Mortality Outcomes of Heart Failure After Acute Myocardial Infarction: A Population-Based Study of 20 812 Patients With First Acute Myocardial Infarction in Western Australia. *Journal of the American Heart Association*.

[3] Abbate A., Biondi-Zoccai G. G. L., Bussani R. (2003). Increased myocardial apoptosis in patients with unfavorable left ventricular remodeling and early symptomatic post-infarction heart failure. *Journal of the American College of Cardiology*.

[4] Baldi A., Abbate A., Bussani R. (2002). Apoptosis and post-infarction left ventricular remodeling. *Journal of Molecular and Cellular Cardiology*.

[5] Olivetti G., Quaini F., Sala R. (1996). Acute myocardial infarction in humans is associated with activation of programmed myocyte cell death in the surviving portion of the heart. *Journal of Molecular and Cellular Cardiology*.

[6] Wiley S. R., Schooley K., Smolak P. J. (1995). Identification and characterization of a new member of the TNF family that induces apoptosis. *Immunity*.

[7] Suda T., Takahashi T., Golstein P., Nagata S. (1993). Molecular cloning and expression of the fas ligand, a novel member of the tumor necrosis factor family. *Cell*.

[8] Suliman A., Lam A., Datta R., Srivastava R. K. (2001). Intracellular mechanisms of TRAIL: Apoptosis through mitochondrial-dependent and -independent pathways. *Oncogene*.

[9] Peter M. E., Krammer P. H. (1998). Mechanisms of CD95 (APO-1/Fas)-mediated apoptosis. *Current Opinion in Immunology*.

[10] Secchiero P., Corallini F., Ceconi C. (2009). Potential prognostic significance of decreased serum levels of TRAIL after acute myocardial infarction. *PLoS ONE*.

[11] Osmancik P., Teringova E., Tousek P., Paulu P., Widimsky P. (2013). Prognostic Value of TNF-Related Apoptosis Inducing Ligand (TRAIL) in Acute Coronary Syndrome Patients. *PLoS ONE*.

[12] Kawakami H., Shigematsu Y., Ohtsuka T. (1998). Increased circulating soluble form of Fas in patients with dilated cardiomyopathy. *Japanese Circulation Journal*.

[13] Tsutamoto T., Wada A., Maeda K. (2001). Relationship between plasma levels of cardiac natriuretic peptides and soluble Fas: Plasma soluble fas as a prognostic predictor in patients with congestive heart failure. *Journal of Cardiac Failure*.

[14] Niessner A., Hohensinner P. J., Rychli K. (2009). Prognostic value of apoptosis markers in advanced heart failure patients. *European Heart Journal*.

[15] Volpato S., Ferrucci L., Secchiero P. (2011). Association of tumor necrosis factor-related apoptosis-inducing ligand with total and cardiovascular mortality in older adults. *Atherosclerosis*.

[16] Anversa P., Cheng W., Liu Y., Leri A., Redaelli G., Kajstura J. (1998). Apoptosis and myocardial infarction. *Basic Research in Cardiology*.

[17] Ferrari R., Guardigli G., Mele D., Percoco G. F., Ceconi C., Curello S. (2004). Oxidative stress during myocardial ischaemia and heart failure. *Current Pharmaceutical Design*.

[18] Steg P. G., James S. K. (2012). Task Force on the management of ST-segment elevation acute myocardial infarction of the European Society of Cardiology (ESC) ESC Guidelines for the management of acute myocardial infarction in patients presenting with ST-segment elevation. *Russian Journal of Cardiology*.

[19] Feldman L. J., Mazighi M., Scheuble A. (2001). Differential expression of matrix metalloproteinases after stent implantation and balloon angioplasty in the hypercholesterolemic rabbit. *Circulation*.

[20] Secchiero P., Gonelli A., Corallini F., Ceconi C., Ferrari R., Zauli G. (2010). Metalloproteinase 2 cleaves in vitro recombinant TRAIL: potential implications for the decreased serum levels of trail after acute myocardial infarction. *Atherosclerosis*.

[21] Ashkenazi A., Pai R. C., Fong S. (1999). Safety and antitumor activity of recombinant soluble Apo2 ligand. *The Journal of Clinical Investigation*.

[22] Jo M., Kim T.-H., Seol D.-W. (2000). Apoptosis induced in normal human hepatocytes by tumor necrosis factor- related apoptosis-inducing ligand. *Nature Medicine*.

[23] Li J. H., Kirkiles-Smith N. C., McNiff J. M., Pober J. S. (2003). Trail induces apoptosis and inflammatory gene expression in human endothelial cells. *The Journal of Immunology*.

[24] Ehrlich S., Infante-Duarte C., Seeger B., Zipp F. (2003). Regulation of soluble and surface-bound TRAIL in human T cells, B cells, and monocytes. *Cytokine*.

[25] LeBlanc H. N., Ashkenazi A. (2003). Apo2L/TRAIL and its death and decoy receptors. *Cell Death & Differentiation*.

[26] Toffoli B., Bernardi S., Candido R., Zacchigna S., Fabris B., Secchiero P. (2012). TRAIL shows potential cardioprotective activity. *Investigational New Drugs*.

[27] Secchiero P., Candido R., Corallini F. (2006). Systemic tumor necrosis factor-related apoptosis-inducing ligand delivery shows antiatherosclerotic activity in apolipoprotein E-null diabetic mice. *Circulation*.

[28] Hallén J., Jensen J. K., Fagerland M. W., Jaffe A. S., Atar D. (2010). Cardiac troponin I for the prediction of functional recovery and left ventricular remodelling following primary percutaneous coronary intervention for ST-elevation myocardial infarction. *Heart*.

[29] Mayr A., Mair J., Klug G. (2011). Cardiac troponin T and creatine kinase predict mid-term infarct size and left ventricular function after acute myocardial infarction: A cardiac MR study. *Journal of Magnetic Resonance Imaging*.

[30] Ohtsuka T., Hamada M., Sasaki O. (1999). Clinical implications of circulating soluble Fas and Fas ligand in patients with acute myocardial infarction. *Coronary Artery Disease*.

[31] Soeki T., Tamura Y., Shinohara H., Sakabe K., Onose Y., Fukuda N. (2003). Relation between circulating soluble Fas ligand and subsequent ventricular remodelling following myocardial infarction. *Heart*.

[32] Nilsson L., Szymanowski A., Swahn E., Jonasson L. (2013). Soluble TNF Receptors Are Associated with Infarct Size and Ventricular Dysfunction in ST-Elevation Myocardial Infarction. *PLoS ONE*.

